# Duck TRIM35 Promotes Tembusu Virus Replication by Interfering with RIG-I-Mediated Antiviral Signaling in Duck Embryo Fibroblasts

**DOI:** 10.1128/spectrum.03858-22

**Published:** 2022-11-29

**Authors:** Peng Zhou, Huijun Zheng, Aixin Liu, Wanrong Wu, Qingxiang Zhang, Hui Jin, Qigai He, Rui Luo

**Affiliations:** a State Key Laboratory of Agricultural Microbiology, College of Veterinary Medicine, Huazhong Agricultural University, Wuhan, Hubei, China; b Key Laboratory of Preventive Veterinary Medicine in Hubei Province, the Cooperative Innovation Center for Sustainable Pig Production, Wuhan, Hubei, China; Thomas Jefferson University

**Keywords:** DTMUV, duRIG-I, duTRIM35, innate immunity, ubiquitination

## Abstract

In China, the duck industry has been severely impacted by the newly emerging duck Tembusu virus (DTMUV). For DTMUV to successfully infect host cells, it employs several strategies that subvert the host's innate immune response. It has been found that several viral proteins encoded by DTMUV have strategically targeted the crucial molecules of the RIG-I-like Receptor (RLR) signaling pathway to antagonize host antiviral responses. However, it is not well known how the host proteins manipulated by DTMUV contribute to innate immune evasion. The present study reports that duck TRIM35 (duTRIM35) antagonizes DTMUV-induced innate immune responses by targeting duck RIG-I (duRIG-I) in duck embryo fibroblasts. A significant increase in duTRIM35 expression occurred during DTMUV infection. DuTRIM35 overexpression suppressed DTMUV-triggered expression of interferon beta (IFN-β) and interferon-stimulated genes (ISGs), promoting viral replication, whereas knockdown of duTRIM35 augments the innate immune response, reducing viral replication. Furthermore, duTRIM35 significantly impaired the IFN-β expression mediated by duRIG-I but not by other RLR signaling molecules. Mechanistically, duTRIM35 interfered with duRIG-I-duTRIM25 interaction and impeded duTRIM25-mediated duRIG-I ubiquitination by interacting with both duRIG-I and duTRIM25. Our findings indicate that duTRIM35 expression induced by DTMUV infection interfered with the duRIG-I-mediated antiviral response, illustrating a novel strategy in which DTMUV can evade the host's innate immunity.

**IMPORTANCE** Duck Tembusu virus (DTMUV), an emerging flavivirus pathogen causing a substantial drop in egg production and severe neurological disorders in duck populations, has led to massive economic losses in the global duck industry. DTMUV has employed various strategies to subvert the host's innate immune response to establish a productive infection in host cells. In this study, we report that duck TRIM35 (duTRIM35) expression was upregulated upon DTMUV infection *in vitro* and *in vivo*, and its expression antagonized DTMUV-induced innate immune responses by targeting duck RIG-I (duRIG-I) in duck embryo fibroblasts. Further studies suggest that duTRIM35 interfered with duRIG-I-duTRIM25 interaction and impeded duTRIM25-mediated duRIG-I ubiquitination by interacting with both duRIG-I and duTRIM25. Together, these results revealed that duTRIM35 expression induced by DTMUV infection downregulated duRIG-I-mediated host antiviral response, which elucidated a novel strategy of DTMUV for innate immune evasion.

## INTRODUCTION

Tembusu virus (TMUV), an emerging *Flavivirus* in the family *Flaviviridae* (1), causes severe egg drop syndrome and fatal encephalitis in domestic ducks. A severe duck TMUV (DTMUV) outbreak in eastern China in 2010 spread to most duck-producing regions of China, Malaysia, and Thailand, resulting in substantial economic losses (2–4). This virus has a positive-sense, single-stranded RNA genome approximately 11 kb in size containing an open reading frame (ORF) (5, 6). The ORF encodes one large precursor polyprotein, and this precursor polyprotein is cleaved immediately by viral and host cell proteases into three structural proteins (capsid, precursor membrane, and envelope glycoprotein) and seven nonstructural proteins (NS1, NS2A, NS2B, NS3, NS4A, NS4B, and NS5) (6–8). Accumulating evidence suggests that the structural proteins contribute mainly to viral entry and virion assembly, while NS proteins play pivotal roles in viral replication and modulation of the host's innate immunity (9–11).

The host's innate immune response to pathogen-associated molecular patterns (PAMPs) is the first line of defense against invading pathogens (12, 13). During flavivirus infection, the incoming virus is recognized by the cytoplasmic retinoic acid-inducible gene I (RIG-I)-like receptors (RLRs), including RIG-I as well as the melanoma differentiation-associated gene 5 (MDA5). RIG-I contains tandem N-terminal caspase activation and recruitment domains (CARDs), a DEAD/H-box helicase domain, and a C-terminal RNA-binding domain (CTD) (14, 15). Once the CTD of RIG-I detects viral RNA, RIG-I undergoes conformational changes and activates downstream adaptor mitochondrial antiviral signaling proteins (MAVS) (also known as VISA, IPS-1, and Cardif) (16–19). Subsequently, MAVS recruits the TBK1-IKKε complexes, which drive the activation of IRF3/7 and NF-κB, resulting in the production of interferon beta (IFN-β) and IFN-stimulated genes (ISGs) (20, 21).

Although RIG-I is a crucial receptor to trigger innate immune responses, different flaviviruses have developed various mechanisms to attenuate its activity to evade host immune responses. For instance, Zika virus (ZIKV) NS5 protein hampers the K63-linked polyubiquitination of RIG-I by interacting with RIG-I, thus dampening RIG-I-mediated signaling (22). Previous reports showed that West Nile virus (WNV) NS1 interferes with IFN-β production by interacting with MDA5/RIG-I, subsequently leading to proteasomal degradation (23). As with other flaviviruses, several reports have shown that DTMUV employs different mechanisms to attenuate IFN-β signaling by targeting RIG-I. DTMUV NS1 has been reported to inhibit the antiviral responses by weakening the association between RIG-I/MDA5 and VISA in HEK-293 cells (24). In addition to subverting the RLR signaling by viral proteins, DTMUV can modulate host factor expression to attenuate the RLR-mediated antiviral response and facilitate replication. Our previous studies found that DTMUV upregulates the duck IFI35 expression that impedes the double-stranded RNA (dsRNA) recognition by duRIG-I by directly interacting with duRIG-I (25). A recent study reported that the duRIG-I-mediated signaling pathway was blocked by LGP2 during DTMUV infection, suppressing the IFN-β production and promoting DTMUV replication (26).

The tripartite motif-containing (TRIM) proteins are found in all vertebrates including avian, most of which contain a conserved RING domain plus one or two B-boxes and an N-terminal coiled-coil (CC) domain (27). By their RING domains, TRIM proteins can act as E3 ligases, which promote the ubiquitination of a wide range of target proteins (28). There is extensive evidence that TRIM proteins modulate innate immunity and regulate viral replication in the host. For example, Japanese encephalitis virus (JEV) infection promotes TRIM21 expression to downregulate IRF3 phosphorylation and inhibits interferon-β production in human microglia cells (29). Epstein-Barr virus (EBV) employs TRIM29 to attenuate cyclic GMP-AMP synthase (cGAS)-STING signaling by inducing ubiquitin-proteasomal degradation of STING (30). Human TRIM35 restricts influenza A virus (IAV) infection by triggering the proteasomal degradation of IAV PB2, hence impeding the suppression of TRAF3 activation by PB2 (31). Fish TRIM35 promotes grouper nodavirus replication by inhibiting the MAVS-, MITA-, or TBK1-mediated innate immunity (32). In this study, we demonstrate that DTMUV infection impairs duck RLR-mediated antiviral signaling and facilitates viral replication by inducing duck TRIM35 (duTRIM35) expression. A specific finding was that duTRIM35 interacted with duRIG-I and inhibited duTRIM25's ability to perform duRIG-I K63-linked polyubiquitination, leading to the inhibition of IFN-β production. As a result of our research, a novel mechanism has been discovered by which DTMUV escapes the host's innate immune response.

## RESULTS

### DTMUV infection induces the expression of duTRIM35.

**FIG 1:**
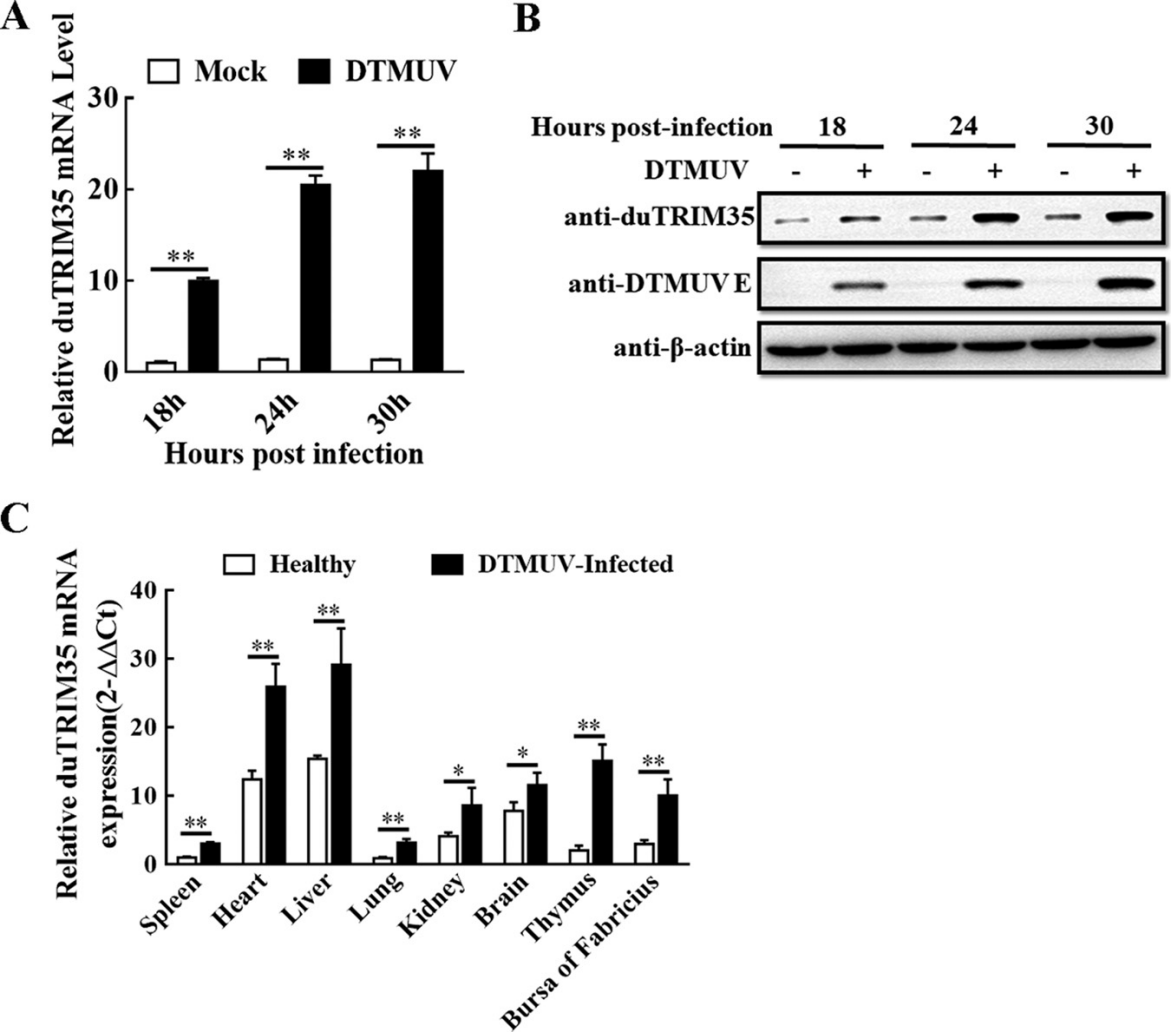
DTMUV infection induces the expression of duTRIM35. (A and B) DEFs were inoculated with DTMUV at a multiplicity of infection (MOI) of 1 for the indicated time points. qRT-PCR and Western blotting were used to measure duTRIM35 protein and mRNA levels. (C) Quantitative analysis of duTRIM35 mRNA in healthy and DTMUV-infected duck tissues. Relative expression levels of duTRIM35 mRNA were determined using qRT-PCR. The standard deviations of the means from three different experiments were calculated and displayed as error bars. *, *P *< 0.05; **, *P *< 0.01 (unpaired Student's *t* test).

To assess the role of duTRIM35 in modulating DTMUV-induced innate immunity, we analyzed its expression in duck embryo fibroblasts (DEFs) at different time points following infection with DTMUV. Significant increases in protein abundance and mRNA expression levels of duTRIM35 were observed during DTMUV infection (Fig. 1A and B). Further analysis of duTRIM35 expression *in vivo* was performed by quantitative real-time PCR (qRT-PCR) to determine the transcription levels of duTRIM35 mRNA in different tissues from healthy or DTMUV-infected ducks. As illustrated in Fig. 1C, tissues infected with DTMUV, including the spleen, heart, liver, kidney, brain, thymus, and bursa of Fabricius, displayed ubiquitously increased expression of duTRIM35. Notably, the expression of duTRIM35 mRNAs increased highest in the thymus after DTMUV infection, reaching 7.27-fold compared to the healthy tissues. These findings suggest that, *in vitro* and *in vivo*, DTMUV infection significantly induces the expression of duTRIM35.

### DuTRIM35 promotes DTMUV replication in DEFs.

**FIG 2:**
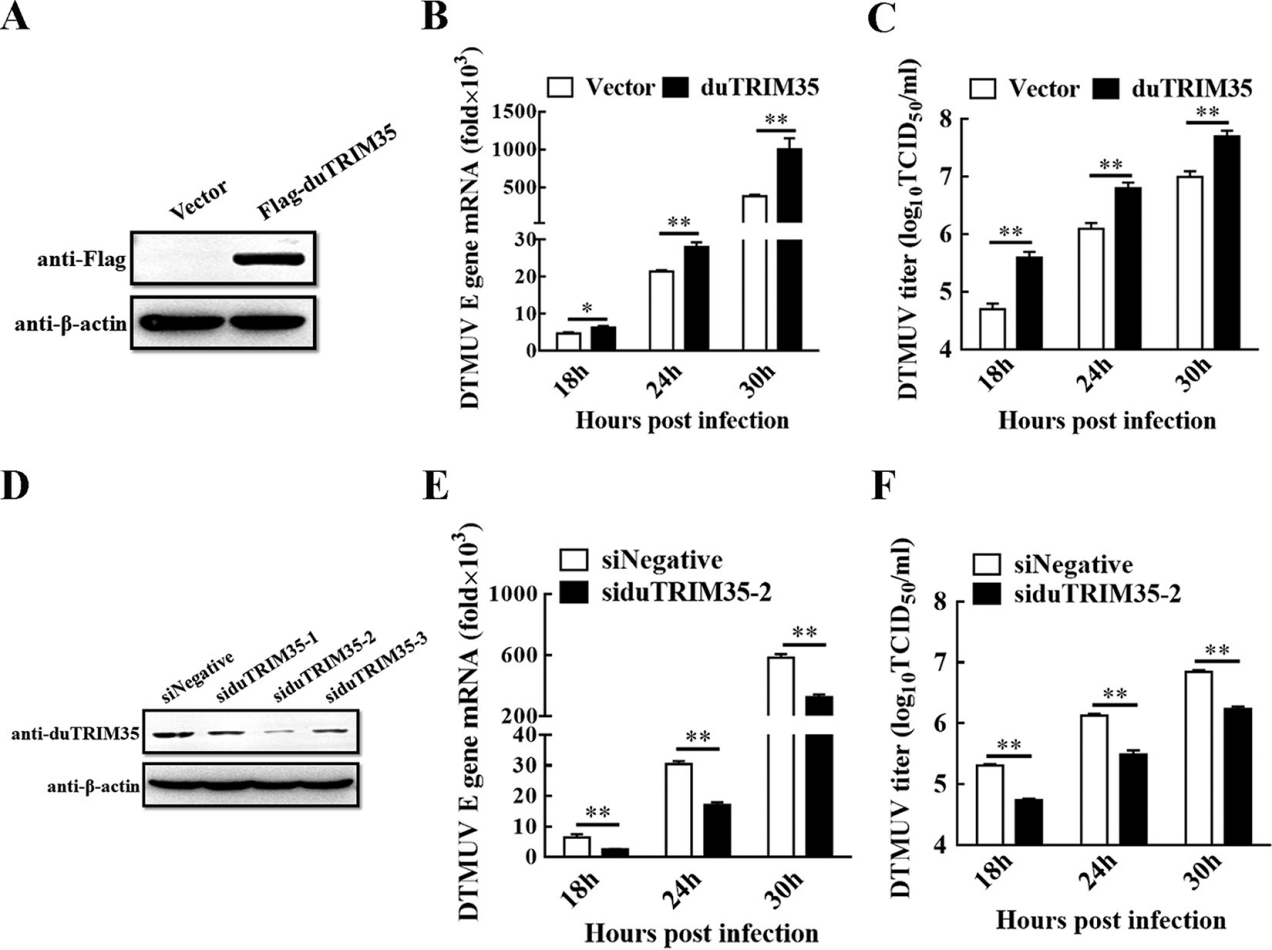
DuTRIM35 promotes DTMUV replication in DEFs. (A) DEFs were transfected with Flag-duTRIM35 or an empty vector. Following transfection for 28 h, cells were collected for immunoblot analysis using the indicated antibodies. (B and C) DEFs were transfected with Flag-duTRIM35 or an empty vector. Following transfection for 24 h, cells were inoculated with DTMUV at an MOI of 1. The viral RNA and titer were measured by qRT-PCR (B) and 50% tissue culture infective dose (TCID_50_) (C). (D) DEFs were transfected with control siRNA (siNegative) and siRNA targeting duTRIM35. Following transfection for 30 h, intracellular proteins were extracted to determine the expression level of duTRIM35 using Western blotting. (E and F) DEFs were transfected with the siduTRIM35-2 or siNegative. Following transfection for 24 h, cells were inoculated with DTMUV at an MOI of 1. The viral RNA and titer were measured by qRT-PCR (E) and TCID_50_ (F). The standard deviations of the means from three different experiments were calculated and displayed as error bars. *, *P *< 0.05; **, *P *< 0.01 (unpaired Student's *t* test).

We first confirmed the expression of the duTRIM35 eukaryotic expression plasmid in DEFs by Western blotting (Fig. 2A). To determine the effect of duTRIM35 on DTMUV replication, the plasmid encoding duTRIM35 was transfected into DEFs followed by DTMUV infection. As shown in Fig. 2B and C, compared to the empty vector group, higher levels of viral RNA and titer were observed in duTRIM35-expressing cells at 18, 24, and 30 h postinfection (hpi), suggesting that overexpression of duTRIM35 promoted DTMUV replication in DEFs. To investigate the function of endogenous duTRIM35 in DTMUV replication, we synthesized three small interfering RNAs (siRNAs) targeting different regions of duTRIM35 mRNA, respectively. The Western blotting results showed that siduTRIM35-2 greatly decreased the expression level of endogenous duTRIM35 (Fig. 2D). Therefore, siduTRIM35-2 was selected for the subsequent experiments. We next examined the effect of duTRIM35 knockdown on DTMUV replication. As illustrated in Fig. 2E and F, a knockdown of duTRIM35 significantly reduced the amount of viral RNA and viral titers at different time points following DTMUV infection. These findings demonstrate that DTMUV replication is promoted by duTRIM35 expression in DEFs.

### DuTRIM35 inhibits the expression of IFN-β and ISGs during DTMUV infection.

**FIG 3:**
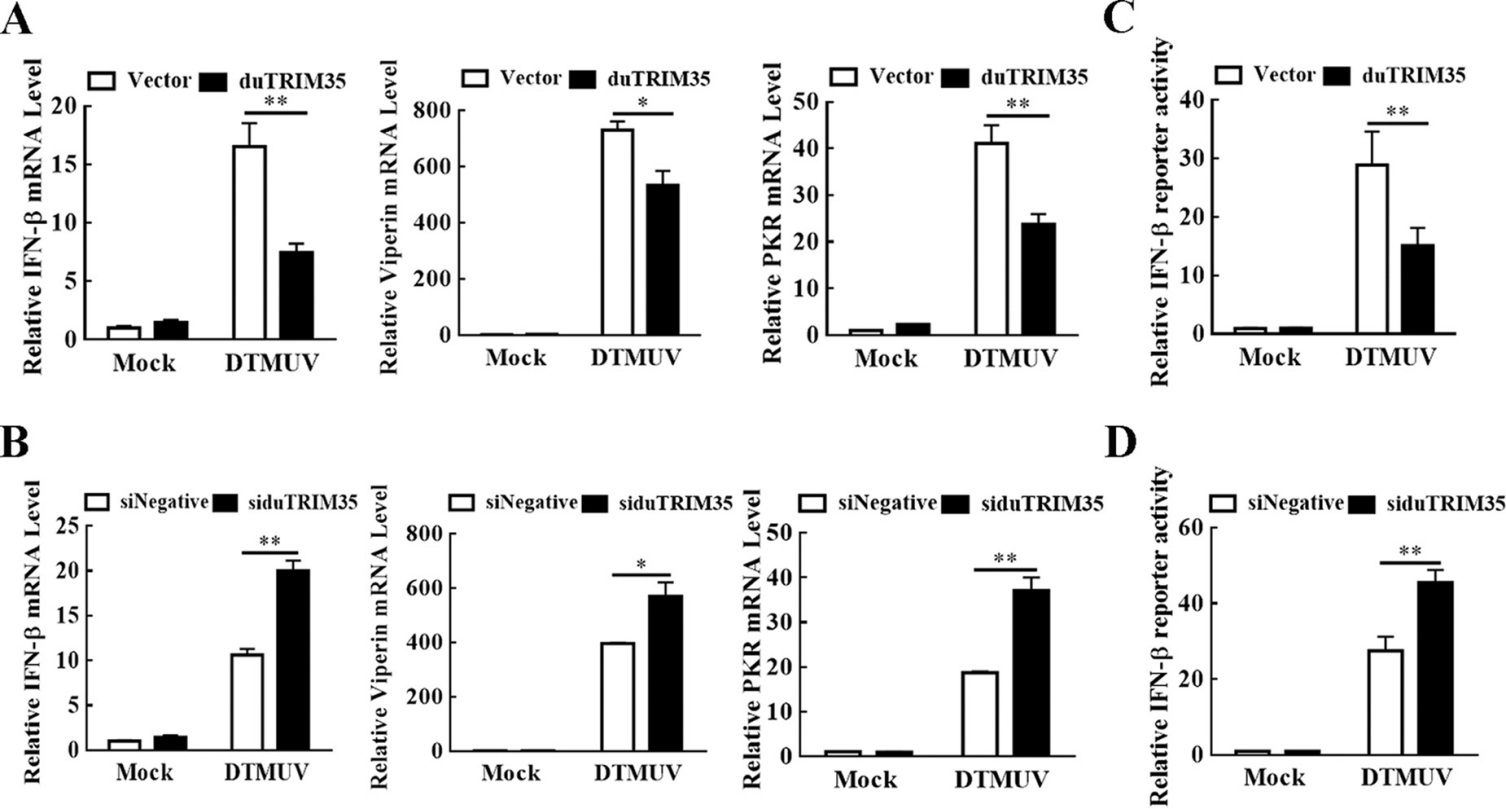
DuTRIM35 inhibits IFN-β and ISG expression during DTMUV infection. (A and B) DEFs were transiently transfected with Flag-duTRIM35 (A) or siduTRIM35 (B) for 24 h and then inoculated with DTMUV at an MOI of 1. Cells were collected to detect IFN-β, viperin, and PKR mRNA by qRT-PCR. (C and D) DEFs were transfected with the reporter plasmids IFN-β-Luc and pRL-TK, together with Flag-duTRIM35 (C) or siduTRIM35 (D) for 24 h and then inoculated with DTMUV at an MOI of 1. The luciferase activities were determined by dual-luciferase reporter assays. The standard deviations of the means from three different experiments were calculated and displayed as error bars. *, *P *< 0.05; **, *P *< 0.01 (unpaired Student's *t* test).

To determine whether duTRIM35 promotes DTMUV replication by modulating the host's innate immunity, the mRNA expression levels of IFN-β, viperin, and PKR mRNA in the duTRIM35-expressing DEFs were detected using qRT-PCR. We observed that overexpression of duTRIM35 downregulated the DTMUV-induced mRNA expression of IFN-β, viperin, and PKR (Fig. 3A). In contrast, knockdown of duTRIM35 dramatically increased the production of IFN-β, viperin, and PKR upon DTMUV infection in DEFs (Fig. 3B). Additionally, luciferase experiments revealed that duTRIM35 overexpression significantly attenuated IFN-β promoter activity triggered by DTMUV (Fig. 3C), whereas knockdown of duTRIM35 expression resulted in the enhancement of IFN-β promoter activity triggered by DTMUV (Fig. 3D). These findings suggest that duTRIM35 expression suppresses IFN-β and ISG expression during DTMUV infection.

### DuTRIM35 interferes with IFN-β production by targeting duRIG-I.

**FIG 4:**
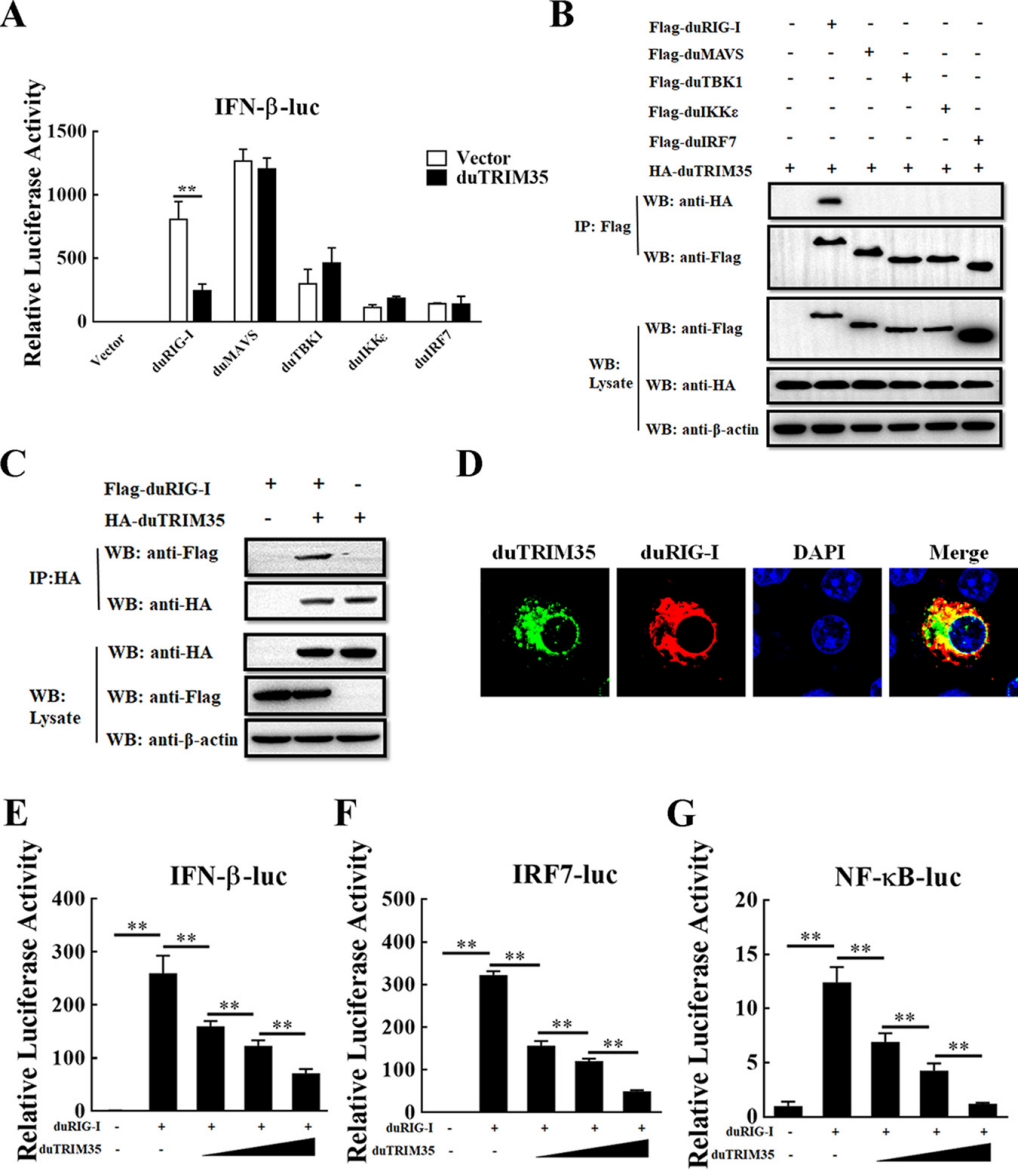
DuTRIM35 interferes with IFN-β production via targeting duRIG-I. (A) DEFs were transiently transfected with HA-duTRIM35, IFN-β-Luc, and pRL-TK, along with the plasmids expressing Flag-duRIG-I, duMAVS, duTBK1, duIKKε, and duIRF7 or empty vector. Luciferase assays were performed at 30 h posttransfection. (B) HEK-293T cells were cotransfected with HA-duTRIM35 and Flag-tagged duRIG-I, duMAVS, duTBK1, duIKKε, or duIRF7. At 28 h posttransfection, cell lysates were immunoprecipitated with indicated antibodies. (C) HEK-293T cells were cotransfected with HA-duTRIM35 and Flag-duRIG-I. At 28 h posttransfection, cell lysates were immunoprecipitated with indicated antibodies. (D) HeLa cells were cotransfected with HA-duTRIM35 and Flag-duRIG-I. At 28 h posttransfection, the cells were collected for IFA to detect duRIG-I protein (red) and duTRIM35 (green) using indicated antibodies. (E to G) DEFs were cotransfected with increasing amounts of HA-duTRIM35 and Flag-duRIG-I, along with pRL-TK and IFN-β-Luc (E), IRF7-Luc (F), or NF-κB-Luc (G). Luciferase assays were carried out 30 h after transfection. The standard deviations of the means from three different experiments were calculated and displayed as error bars. *, *P *< 0.05; **, *P *< 0.01 (unpaired Student's *t* test).

To further determine the target of duTRIM35 in the IFN production signaling, plasmids encoding essential molecules in RLR signaling (including duRIG-I, duMAVS, duTBK1, duIKKε, and duIRF7) and duTRIM35, along with the IFN-β luciferase reporter plasmid, were cotransfected into DEFs. As illustrated in Fig. 4A, overexpression of duTRIM35 significantly inhibited IFN-β promoter activity induced by duRIG-I, whereas IFN-β promoter activity induced by other signal molecules was not attenuated by duTRIM35. Coimmunoprecipitation (Co-IP) assays showed that duTRIM35 specifically binds to duRIG-I (Fig. 4B). Additionally, the duTRIM35-duRIG-I interaction was further confirmed using anti-hemagglutination (HA) antibodies in a reverse Co-IP (Fig. 4C). The confocal microscopy results further demonstrated that duTRIM35 and duRIG-I were predominately colocalized in the cytoplasm (Fig. 4D). Moreover, we observed that duTRIM35 dose dependently inhibited the IFN-β, IRF7, and NF-κB promoter activities mediated by duRIG-I (Fig. 4E to G). Altogether, these findings indicate that duTRIM35 blocks the production of duck IFN-β by targeting duRIG-I.

### DuTRIM35 interacts with duRIG-I through its RING domain.

**FIG 5:**
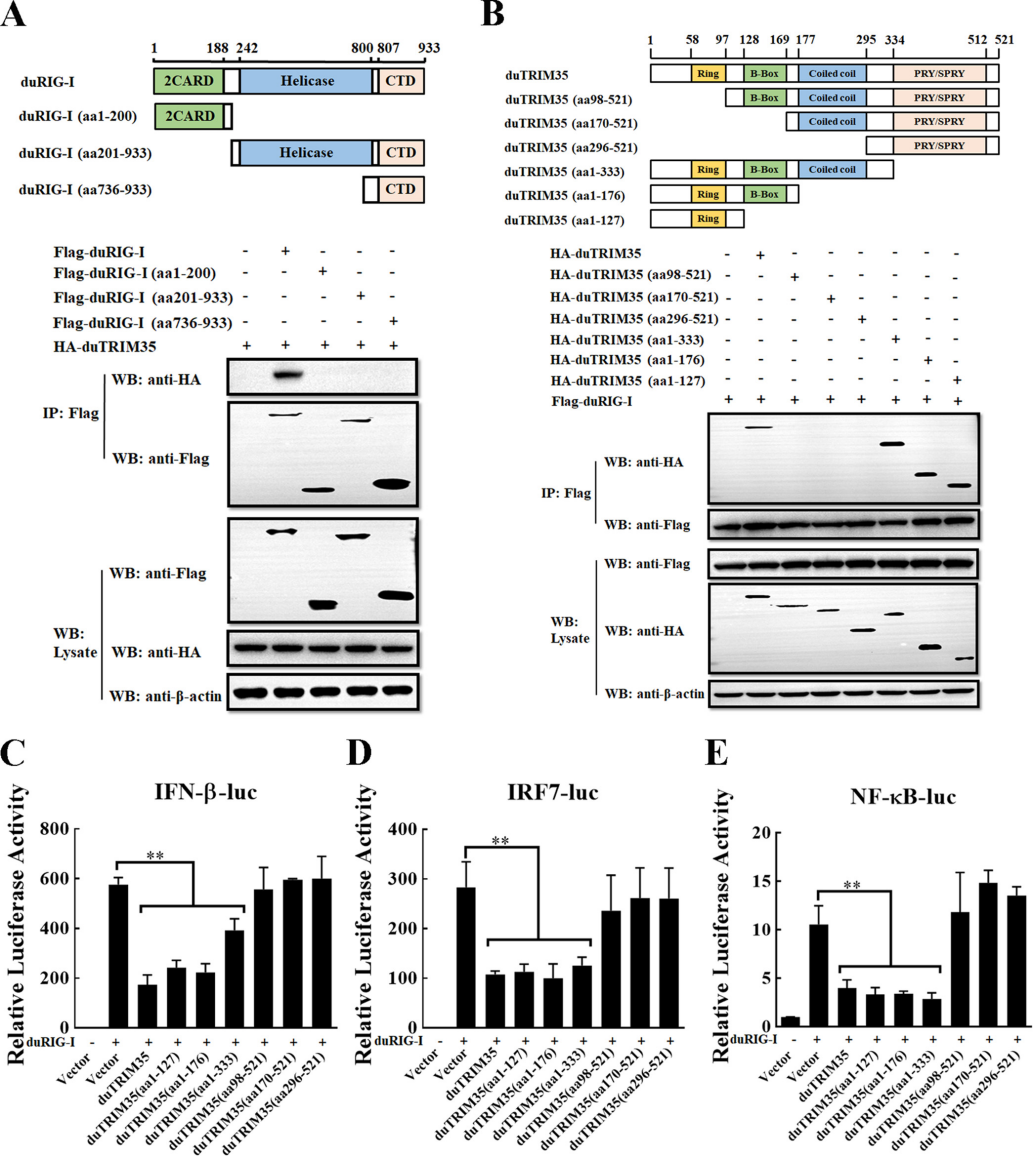
DuTRIM35 binds to duRIG-I through its RING domain. (A) HEK-293T cells were cotransfected with HA-duTRIM35 and Flag-duRIG-I or its truncation mutants. After transfection for 28 h, cell lysates were immunoprecipitated with anti-Flag antibodies. (B) HEK-293T cells were cotransfected with Flag-duRIG-I and HA-duTRIM35 or its truncation mutants. After transfection, cell lysates were immunoprecipitated with anti-Flag antibodies. (C to E) DEFs were cotransfected with Flag-duRIG-I and HA-duTRIM35 or its truncation mutants, along with pRL-TK and IFN-β-Luc (C), IRF7-Luc (D), or NF-κB-Luc (E). The activity of IFN-β/IRF7/NF-κB-Luc was measured at 30 h posttransfection. The standard deviations of the means from three different experiments were calculated and displayed as error bars. *, *P *< 0.05; **, *P *< 0.01 (unpaired Student's *t* test).

To identify which domain of duRIG-I interacts with duTRIM35, we cotransfected the plasmids encoding the Flag-tagged duRIG-I deletion mutants and HA-tagged duTRIM35 in HEK-293T cells. Through Co-IP assays, we found that duTRIM35 was coprecipitated with full-length duRIG-I but not with its deletion mutants (Fig. 5A), suggesting that the integrity of duRIG-I is required for its binding to duTRIM35. To further ascertain the crucial regions of duTRIM35 responsible for the association with duRIG-I, HA-tagged duTRIM35 deletion mutants and Flag-tagged duRIG-I were cotransfected into HEK-293T cells. Co-IP experiments showed that deletion of the RING domain of duTRIM35 abrogated its interaction with duRIG-I (Fig. 5B). Notably, all deletion mutants of duTRIM35 containing the RING domain could effectively attenuate the activity of IFN-β, IRF7, and NF-κB promoters induced by duRIG-I (Fig. 5C to E). These data indicate that the RING domain of duTRIM35 is essential for its interaction with duRIG-I and attenuation of duRIG-I-mediated IFN-β production.

### DuTRIM35 impairs the K63-linked polyubiquitination of duRIG-I.

**FIG 6:**
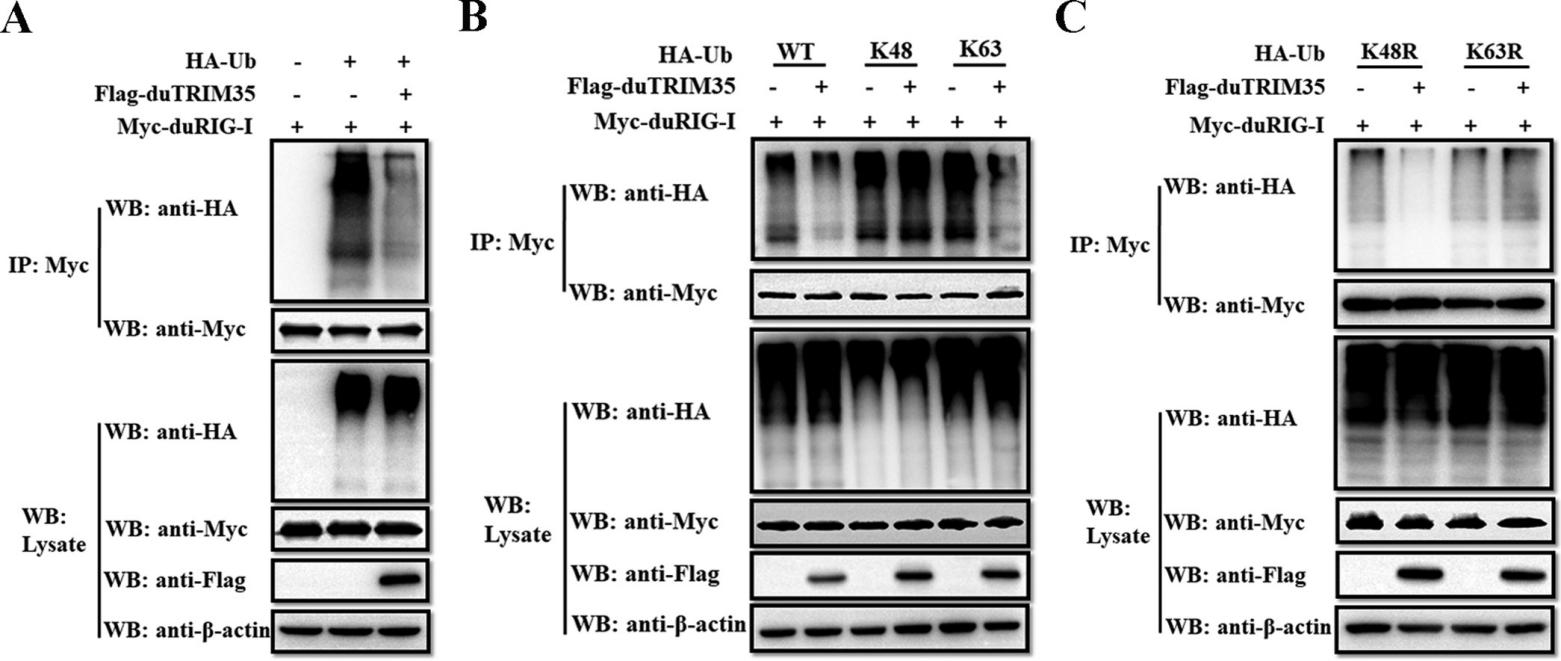
DuTRIM35 interfered with the K63-linked polyubiquitination of duRIG-I. (A to C) DEFs were cotransfected with Myc-tagged duRIG-I and Flag-tagged duTRIM35, together with HA-Ub (A), HA-K48/K63-Ub (B), or HA-K48R/K63R-Ub (C). At 28 h posttransfection, cell lysates were immunoprecipitated with anti-Myc antibodies.

Since the K63-linked ubiquitination of RIG-I is essential for triggering the antiviral responses (33), we investigated whether duRIG-I ubiquitination was regulated by duTRIM35. Lysates prepared from DEFs cotransfected with Myc-duRIG-I, HA-ubiquitin (HA-Ub), and Flag-duTRIM35 were immunoprecipitated with anti-Myc antibodies and examined the polyubiquitination levels of duRIG-I by immunoblotting. As shown in Fig. 6A, the ubiquitination of duRIG-I was substantially suppressed in the cells expressing duTRIM35. To further dissect the polyubiquitination type of duRIG-I mediated by duTRIM35, we coexpressed Myc-duRIG-I, Flag-duTRIM35, and HA-tagged wild-type ubiquitin (HA-Ub) or ubiquitin mutants containing arginine substitutions on all lysine residues except the lysine at position 48 (HA-Ub-K48) or 63 residues (HA-Ub-K63) in DEFs. Immunoprecipitation assays showed that the duTRIM35 significantly reduced the K63-linked rather than K48-linked ubiquitination of duRIG-I (Fig. 6B). To further verify the results, we coexpressed Myc-duRIG-I with HA-tagged ubiquitin mutants harboring a single lysine to arginine substitution at position 48 (HA-Ub-K48R) or 63 (HA-Ub-K63R) residues in the absence or presence of Flag-duTRIM35 in DEFs. As shown in Fig. 6C, duTRIM35 overexpression markedly impaired duRIG-I polyubiquitination in the presence of the K48R mutant but not the K63R mutant. These data indicate that duTRIM35 specifically suppresses the K63-linked polyubiquitination of duRIG-I.

### DuTRIM35 attenuates duTRIM25-induced duRIG-I ubiquitination.

**FIG 7:**
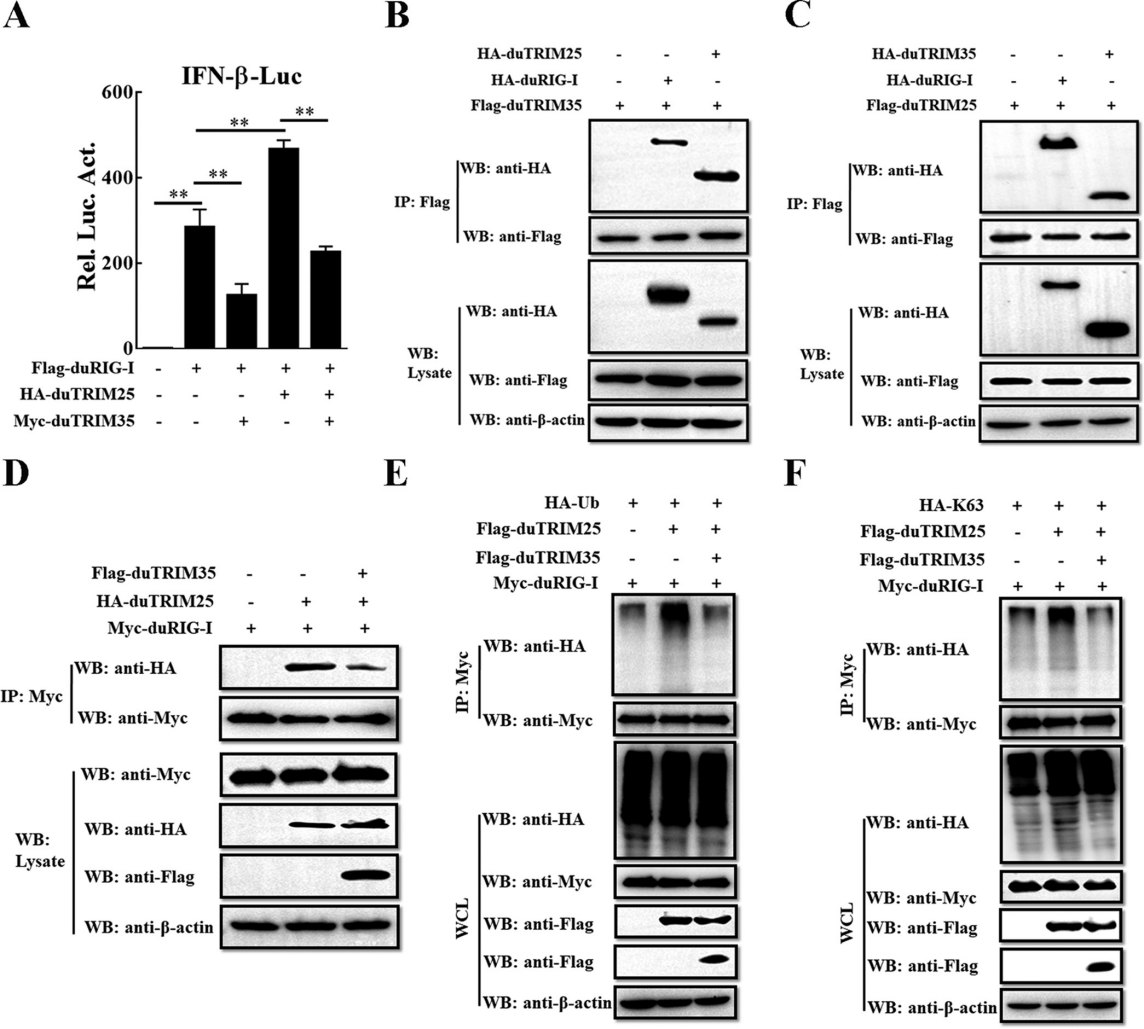
DuTRIM35 attenuates duTRIM25-induced duRIG-I ubiquitination. (A) DEFs were cotransfected with Flag-duRIG-I, pRL-TK, and IFN-β-Luc, along with HA-duTRIM25 and Myc-duTRIM35 or an empty vector. The IFN-β-Luc activity was measured at 30 h posttransfection. (B) Co-IP analysis of the interaction between Flag-duTRIM35 and HA-duTRIM25 or duRIG-I in HEK-293T cells. (C) Co-IP analysis of the interaction between Flag-duTRIM25 and HA-duTRIM35 or duRIG-I in HEK-293T cells. (D) HEK-293T cells were cotransfected with Myc-duRIG-I and HA-duTRIM25, along with Flag-duTRIM35 or an empty vector. After transfection for 28 h, cell lysates were immunoprecipitated with anti-Myc antibodies. (E and F) DEFs were cotransfected with Myc-tagged duRIG-I, Flag-tagged duTRIM25, and HA-Ub (E) or HA-K63-Ub (F), along with Flag-duTRIM35 or an empty vector. After transfection for 28 h, cell lysates were immunoprecipitated with anti-Myc antibodies.

It has been demonstrated that duck TRIM25 contributes to duRIG-I ubiquitination and enhancement of interferon production (34), which promotes us to speculate that duTRIM35 suppressed duRIG-I signaling via targeting duTRIM25. To test this hypothesis, we first investigate the effects of duTRIM35 on IFN-β expression triggered by duTRIM25-duRIG-I signaling. The expression plasmids encoding duRIG-I and duTRIM25 were cotransfected with IFN-β-Luc, pRL-TK, and duTRIM35 in DEFs, and luciferase activities were detected at 30 h after transfection. As illustrated in Fig. 7A, duTRIM25 overexpression markedly enhanced the duRIG-I-induced activation of the IFN-β promoter. However, duTRIM25-mediated enhancement of duRIG-I signaling was considerably impaired in the duTRIM35-expressing cells (Fig. 7A). Since our results suggest that duTRIM25 may be the target of duTRIM35 in blocking duRIG-I signaling, we next determined whether duTRIM35 interacts with duTRIM25. To achieve this, we cotransfected the plasmids encoding Flag-tagged duTRIM35 and HA-tagged duTRIM25 or duRIG-I and performed Co-IP experiments with anti-Flag antibodies. In line with our expectations, duTRIM35 interacted with both duTRIM25 and duRIG-I (Fig. 7B). Additionally, their interactions were further confirmed by a reverse Co-IP experiment (Fig. 7C). Based on these results, it can be concluded that duTRIM35 interacts with both duRIG-I and duTRIM25.

To investigate whether duTRIM35 binding to duTRIM25 and duRIG-I impairs duRIG-I-mediated antiviral signaling, we evaluated the effects of duTRIM35 on duRIG-I-duTRIM25 interaction. For this purpose, we cotransfected the plasmids expressing Myc-tagged duRIG-I and HA-tagged duTRIM25 together with the duTRIM35 expression plasmid. The Co-IP experiments showed that the association between duRIG-I and duTRIM25 was remarkably decreased in the presence of duTRIM35 (Fig. 7D), suggesting that duTRIM35 may downregulate duRIG-I-induced antiviral responses by disrupting the formation of the duRIG-I-duTRIM25 complex. We then investigated the potential influence of duTRIM35 on duTRIM25-mediated duRIG-I ubiquitination. As shown in Fig. 7E, duTRIM25 expression significantly promoted the ubiquitination of duRIG-I. However, the level of duTRIM25-induced duRIG-I ubiquitination was greatly decreased in the presence of duTRIM35 (Fig. 7E). As expected, the duTRIM25-mediated K63-linked polyubiquitination was also reduced in the cells expressing duTRIM35 (Fig. 7F). These results suggest that duTRIM35 inhibits duRIG-I signaling via impairing K63-linked polyubiquitination of duRIG-I induced by duTRIM25.

## DISCUSSION

During flavivirus replication, the viral replication intermediates, such as dsRNA, are recognized by host RLR that triggers type I IFN production and antiviral gene expression. Consequently, most flaviviruses have developed strategies to decrease the IFN expression to facilitate their replication in host cells. For instance, NS2A, NS2B, and NS4B of Zika virus (ZIKV) inhibit MDA5/RIG-I-mediated IFN-β expression via blocking TBK1 phosphorylation, whereas NS4A and NS5 efficiently interfere with RIG-I-induced IRF3 activation (35, 36). Like other flaviviruses, DTMUV-encoded proteins employed several strategies to inhibit RLR antiviral signaling. DTMUV NS1 blocks interferon-β expression by attenuating the association between RIG-I/MDA5 and VISA in HEK-293 cells (24). Recently, we reported that DTMUV NS2B antagonizes IFN-β expression by targeting duck MAVS for degradation (37). Besides combating RLR signaling by viral proteins, DTMUV also induces host factors expression to negatively regulate the RLR antiviral signaling. We previously reported that DTMUV promotes the duIFI35 expression to hinder the dsRNA recognition by duRIG-I via its interaction with duRIG-I (25). Our current study identified a novel host factor, duTRIM35, manipulated by DTMUV to attenuate RLR-mediated innate immune responses. Firstly, we found that duTRIM35 expression was upregulated upon DTMUV infection *in vitro* and *in vivo* (Fig. 1), and its expression facilitated DTMUV replication (Fig. 2). Secondly, DTMUV-triggered IFN-β and ISG production dramatically decreased by overexpression of duTRIM35 (Fig. 3). Thirdly, duTRIM35 inhibited the IFN-β expression mediated by duRIG-I, but not duMAVS, duTBK1, duIKKε, and duIRF7, via directly interacting with duRIG-I (Fig. 4 and 5). Lastly, overexpression of duTRIM35 significantly impaired the duRIG-I-duTRIM25 interaction and attenuated duTRIM25-mediated duRIG-I K63 ubiquitination and activation (Fig. 6 and 7).

TRIM35 was originally discovered as a tumor suppressor with the potential to reduce tumor cell proliferation, clonogenicity, and tumorigenicity (38, 39). Several studies have demonstrated that TRIM35 is essential to the host's innate immune response because it can catalyze the ubiquitination process during viral infection. For example, human and porcine TRIM35s inhibit viral replication by promoting TRAF3 K63-linked polyubiquitination to upregulate IFN-β production (31, 40). Moreover, Huang et al. reported that fish TRIM35 suppresses MITA-, MAVS-, and TBK1-induced innate immune response, thereby promoting virus replication (32). However, it is unclear what role TRIM35 plays in modulating avian innate immunity. In the present study, we revealed that duTRIM35 inhibited the innate antiviral response by attenuating duTRIM25-mediated duRIG-I K63 ubiquitination to facilitate DTMUV replication, suggesting that avian TRIM35 employed a different strategy to regulate the antiviral innate immune responses compared to mammal and fish TRIM35s. Even though duTRIM35 shares similar architecture with human TRIM35, including the N-terminal RBCC domain, and a C-terminal PRY/SPRY domain, duTRIM35 has only 52% amino acid identity with human TRIM35. The sequence differences in TRIM35 between ducks and humans may contribute to its binding of different molecular targets in innate immunity.

RIG-I is an important member of the RLRs family that recognizes viral RNAs and recruits MAVS to activate downstream kinases TBK1 and IKKε, resulting in IRF3 translocation and type I IFN production. Several cellular factors have been reported to downregulate RIG-I activity and suppress RLR-mediated antiviral signaling (41). For example, SEC14L1 binds to the N-terminal domain of RIG-I and blocks the downstream recruitment of MAVS, thus inhibiting IFN-β expression (42). Furthermore, Jounai et al. found that, upon association with the CARDs of both MAVS and RIG-I, the Atg5-Atg12 conjugate inhibits RLR signaling and attenuates type I IFN production (43). However, less is known about the regulation of avian RLR antiviral signaling compared to mammals. Recently, duLGP2 was identified as a host factor to block duRIG-I-mediated antiviral responses after DTMUV infection, suppressing the IFN-β production and promoting viral replication (26). Currently, we revealed that duTRIM35 downregulated duck RLR signaling by interacting with duRIG-I for the first time (Fig. 4). Identifying novel host factors involved in regulating RLR signaling will enrich our understanding of avian innate immunity.

Accumulating research has found that RIG-I activity could be regulated by ubiquitination modification. By catalyzing the process of K63-linked polyubiquitination, E3 ligases, such as TRIM25, Riplet, TRIM4, and MEX3C, positively regulate the activity of RIG-I (44–47). In contrast, the antiviral response is dampened by the K48-linked polyubiquitination and degradation of RIG-I mediated by Siglec-G, RNF125, IFI35, or RNF122 (48–51). Additionally, inhibiting ubiquitin ligases binding with RIG-I is another strategy to interrupt RIG-I signaling. For instance, the N protein encoded by severe acute respiratory syndrome coronavirus (SARS-CoV) or Middle East respiratory syndrome coronavirus (MERS-CoV) directly interacts with TRIM25 to prevent the K63-linked polyubiquitination of RIG-I (52). Moreover, Yang et al. found that overexpression of RTN3 suppresses the RIG-I K63-linked polyubiquitination in a TRIM25-dependent style (53). A previous study has shown that duTRIM25 was upregulated in duck embryo fibroblasts (DEFs) by DTMUV infection, and its expression inhibited DTMUV replication (54). In addition, Miranzo-Navarro and Magor reported that the association between RIG-I and TRIM25 in duck is essential for stimulating IFN-β production (34). These findings indicate that duTRIM25 inhibits DTMUV replication, possibly by enhancing duRIG-I-mediated IFN-β signaling. In this study, we showed that duTRIM35 interacted with duTRIM25 and markedly inhibited the K63-linked polyubiquitination of duRIG-I (Fig. 5 and 6), prompting us to speculate whether duTRIM35 disrupts the duRIG-I signaling cascade by competitively binding to duRIG-I with duTRIM25. As expected, duTRIM35 overexpression significantly hampered the duRIG-I-duTRIM25 interaction (Fig. 7D), indicating that duTRIM35 inhibits duTRIM25-mediated duRIG-I K63-linked polyubiquitination by disturbing the association between duTRIM25 and duRIG-I, thereby interrupting duRIG-I signaling. However, the precise molecular mechanism by which duTRIM35 interrupted duTRIM25 binding to duRIG-I requires further research.

**FIG 8:**
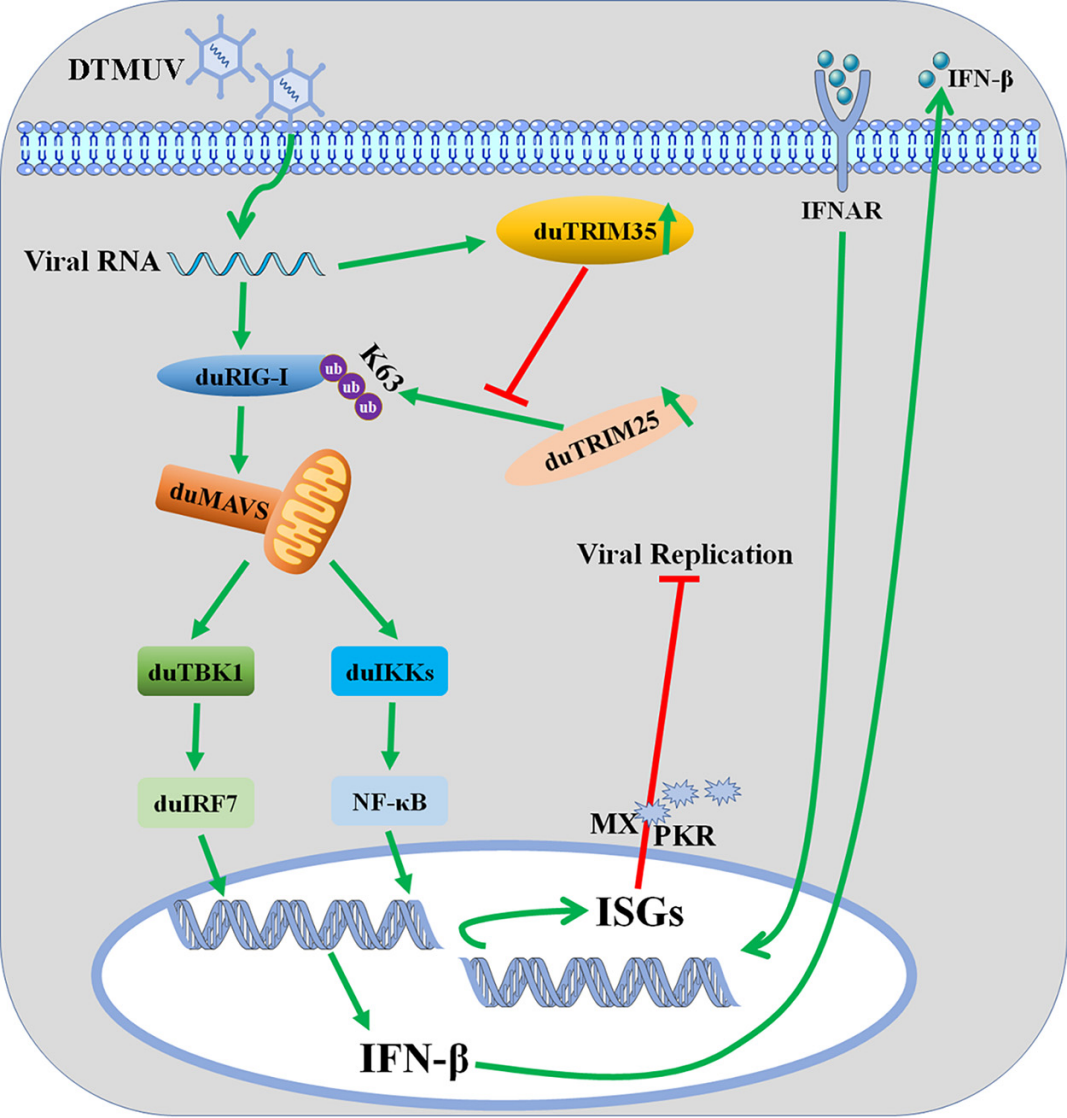
A proposed schematic diagram of the suppression of duRIG-I triggered antiviral innate immune responses by duTRIM35.

In conclusion, we present a schematic model that illustrates how DTMUV inhibits RIG-I signalosome activation to suppress type I interferon production by duTRIM35 (Fig. 8). During DTMUV infection, duRIG-I detects the viral RNA and stimulates the RLR antiviral signaling. To combat the host's innate immunity, DTMUV induces duTRIM35 expression, which interacts with duTRIM25 and suppresses duTRIM25-mediated K63 ubiquitination and activation of duRIG-I by weakening the association between duRIG-I and duTRIM25, thereby attenuating type I interferon expression and facilitating viral replication. Our findings identified a novel host factor manipulated by DTMUV to block the RLR antiviral signaling, which may help develop new antiviral therapeutics to prevent DTMUV infection.

## MATERIALS AND METHODS

### Cell, tissues, viruses, and reagents.

Duck embryo fibroblasts (DEFs) were cultured in minimum essential medium (MEM) (Gibco, USA) containing 10% fetal bovine serum (FBS) (Gibco, USA) at 37°C in a 5% CO_2_ incubator. HeLa and HEK-293T cells were maintained in Dulbecco's modified Eagle's medium (DMEM) (Gibco, USA) supplemented with 10% FBS. DTMUV strain MC (GenBank accession number KX452096) was isolated by our laboratory as previously described (25). Various tissues of three 10-day-old healthy or DTMUV-infected ducklings were obtained from an artificial infection experiment described previously in our lab (55). Tissue samples were snap-frozen into liquid nitrogen and stored at −80°C for RNA isolation. Antibodies against Flag, Myc, HA, and β-actin were obtained from Medical and Biological Laboratories (Nagoya, Japan). Anti-DTMUV E protein monoclonal antibody (MAb) was generated as previously described (25). Mouse anti-duTRIM35 antibody was stocked in our laboratory. Three pairs of small interfering RNA (siRNA) sequences targeting duTRIM35 were synthesized by GenePharma (Shanghai, China), and their sequences are listed in Table S2 in the supplemental material.

### Plasmid construction.

DuTRIM35 and duTRIM25 were amplified from the cDNA of DEFs and subcloned into the pCAGGS expression vector in-frame with an HA or Flag tag at the N terminus. The plasmids encoding duRIG-I, duRIG-I-truncated mutants, duMAVS, duTBK1, duIKKε, and duIRF7 were constructed as previously described (25). DuTRIM35-truncated mutants (amino acids [aa] 1 to 333, aa 1 to 176, aa 1 to 127, aa 98 to 521, aa 170 to 521, and aa 296 to 521) were cloned into the pCAGGS expression vector with an HA tag at the N terminus. The luciferase reporter plasmids including IFN-β-Luc, NF-κB-Luc, and IRF7-Luc have been described previously (56). All plasmid constructs were confirmed by DNA sequencing. The PCR primers used in this study are provided in Table S1 in the supplemental material.

### Real-time PCR analysis.

Total RNA was extracted from DEFs and selected tissues of ducklings using TRIzol reagent (Invitrogen, USA) according to the manufacturer's instructions. RNA was reverse-transcribed to cDNA using the Transcriptor First Strand cDNA synthesis kit (Roche, Switzerland). FastStart Universal SYBR green master mix (Roche, Switzerland) was used in a ViiA 7 system (Applied Biosystems, USA) for quantitative real-time PCR (qRT-PCR). The relative expression levels were determined using the comparative threshold cycle (ΔΔ*C_T_*) method with glyceraldehyde-3-phosphate dehydrogenase (GAPDH) as an internal reference. Quantitative PCR (qPCR) primers employed in this study are listed in Table S1.

### Dual-luciferase reporter assay.

In a 48-well plate, ~80% confluence DEFs were cotransfected using Lipofectamine 2000 (Invitrogen) with a luciferase reporter plasmid (IFN-β-Luc, NF-κB-Luc, or IRF7-Luc) and an internal control pRL-TK, along with appropriate expression plasmids. After transfection, the cells were inoculated with DTMUV for 24 h. Subsequently, the cells were collected, and firefly and *Renilla* luciferase activities were measured through the dual-luciferase reporter assay system (Promega, USA) following the manufacturer's instructions. Representative data were presented as the relative firefly luciferase activities with normalization to the *Renilla* luciferase activities from three independent assays.

### Immunofluorescence and confocal microscopy.

After seeding HeLa cells on coverslips in 24-well plates, the cells were transfected with the expression plasmids. The transfected cells were fixed with 4% paraformaldehyde, permeabilized with 0.1% Triton X-100, and blocked with 5% bovine serum albumin (BSA). Subsequently, the cells were incubated separately with a mouse anti-HA antibody (1:200) or a rabbit anti-Flag antibody (1:50) and then stained with secondary antibodies Alexa Fluor 488-conjugated donkey anti-mouse IgG and Alexa Fluor 594-conjugated donkey anti-rabbit IgG (Invitrogen), followed by treatment with 4′,6-diamidino-2-phenylindole (DAPI) (Invitrogen). Fluorescence was visualized by a Zeiss LSM 880 confocal microscope.

### Western blotting.

Protein samples from the indicated cells were prepared using radioimmunoprecipitation assay (RIPA) buffer (Beyotime) supplemented with 1 mM phenylmethanesulfonyl fluoride (PMSF). The lysates were separated by 12% SDS-PAGE, electroblotted onto polyvinylidene difluoride (PVDF) membranes (Millipore), and then blocked with Tris-buffered saline-Tween (TBST) containing 10% skim milk. The appropriate antibodies were used to probe specific protein bands. An enhanced chemiluminescence (ECL) system (Bio-Rad) was used to detect antibody-antigen complexes.

### Coimmunoprecipitation.

HEK-293T cells were cultured in 10-cm dishes and cotransfected with the protein expression plasmids. After transfection, cells were collected with Co-IP buffer (Beyotime) supplemented with EDTA-free protease inhibitor cocktail (Roche). Cell lysate (0.4 mL) was incubated for each immunoprecipitation with anti-Flag or anti-Myc monoclonal antibodies overnight at 4°C. After further incubation with 40 μL protein A/G plus agarose (Santa Cruz Biotechnology) for 4 h, the beads were collected by centrifugation and washed 4 times with cold IP buffer. Finally, the precipitates were analyzed by standard immunoblot procedures with the appropriate antibody.

### Ubiquitination assay.

DEFs were cotransfected with Myc-duRIG-I, Flag-duTRIM35, or an empty vector in the presence or absence of HA-Ub or its mutations. After transfection, cells were harvested with Co-IP buffer (Beyotime), and cell lysates were immunoprecipitated with anti-Myc MAb, followed by incubating with protein A/G plus-agarose (Santa Cruz Biotechnology). After washing with lysis buffer, the immunoprecipitates were analyzed by standard immunoblot procedures with the appropriate antibodies.

### Statistical analysis.

The data were statistically evaluated using GraphPad Prism software (GraphPad Software, Inc.). The *P* value was determined using an unpaired two-tailed Student's *t* test. A *P* value of <0.05 was regarded as statistically significant, and a *P* value of <0.01 was regarded as highly significant.
