# Supplementary material for: Duck TRIM35 Promotes Tembusu Virus Replication by Interfering with RIG-I-Mediated Antiviral Signaling in Duck Embryo Fibroblasts
Source: Microbiol Spectr. 2022 Nov 29;10(6):e03858-22. doi: 10.1128/spectrum.03858-22 (PMC9769614; doi:10.1128/spectrum.03858-22)
Supplement: Supplemental file 1 — Supplemental material. Download spectrum.03858-22-s0001.pdf, PDF file, 0.2 MB [file spectrum.03858-22-s0001.pdf]

Table S1

Sequences of the primers used for real-time PCR and construction of plasmids

Primer	Sequence (5'-3')
duTRIM35-CDS-F	TATGAATTCATGCTGCCGGCCCCCGCACG
duTRIM35-CDS-R	TATCTCGAGCTACACGGGGGTGTCCTCGTGGATGC
duTRIM35(aa1-333)-R	GCACTCGAGCTAGGTGATGTTGTCCAGCATC
duTRIM35(aa1-176)-R	TATCTCGAGCTAGAAATCCGCAGCCGTCTCCTG
duTRIM35(aa1-127)-R	TATCTCGAGCTAGCCCCGCCGCTGCCCTTC
duTRIM35(aa98-521)-F	GACGAATTCATGGAACCCGCTTCCCCCGACGAC
duTRIM35(aa170-521)-F	GACGAATTCATGCAGGAGACGGCTGCGG
duTRIM35(aa296-521)-F	TATGAATTCATGGCCTGCACGGTGGAGGAG
qduTRIM35-F	AAACACAACCAGGTGGAAGC
qduTRIM35-R	GTCCGACAGCTGCTTGATTT
qduIFNβ-F	ACATCCTTTTGGACACCGACAA
qduIFNβ-R	TTGGACTGCTGAGGATGTTGA
qduViperin-F	TCCAGTTCTGCAAGGAGGAG
qduViperin-R	TTCTTGAACCACCGCTCTCT
qduPKR-F	GTGAAGGTGGTTTTGGGAAT
qduPKR-R	TCACGCTTCACCTTCTCCTTA
qduGAPDH-F	CAAGGCTGAGAATGGGAAACTT
qduGAPDH-R	GCATCTGCCCACTTGATGTT

Table S2

The sequences of siRNAs used in the study.

siRNA	Sequences (5' to 3')
siduTRIM35-1	5' GCACCACGAGGAAGCCAAATT 3'
	5' UUUGGCUUCCUCGUGGUGCTT 3'
siduTRIM35-2	5' GGGCCAAGCUGAAGAACAUTT 3'
	5' AUGUUCUUCAGCUUGGCCCTT 3'
siduTRIM35-3	5' GGAGCCACAUCUACACCUUTT 3'
	5' AAGGUGUAGAUGUGGCUCCTT 3'
siNegative control	5' UUCUCCGAACGUGUCACGUTT 3'
	5' ACGUGACACGUUCGGAGAATT 3'
